# 2-Methoxyestradiol-bis-sulphamate refrains from inducing apoptosis and autophagy in a non-tumorigenic breast cell line

**DOI:** 10.1186/1475-2867-12-37

**Published:** 2012-08-20

**Authors:** Michelle H Visagie, Anna M Joubert

**Affiliations:** 1 Department of Physiology, University of Pretoria, P.O. Box 2034, Pretoria 0001, South Africa

**Keywords:** Apoptosis, Autophagy, Non-tumorigenic, Reactive oxygen species

## Abstract

**Background:**

Anticancer research resulted in the discovery of a promising antimitotic metabolite, 2-methoxyestradiol. 2-Methoxyestradiol-bis-sulphamate, a bis-sulphamoylated analogue exerts antiproliferative- and antimitotic activity. Investigating the anticancer potential of 2-methoxyestradiol-bis-sulphamate requires demonstrating the influence of 2-methoxyestradiol-bis-sulphamate on non-tumorigenic cells. This project focused on the in vitro effects of 2-methoxyestradiol-bis-sulphamate on the non-tumorigenic MCF-12A breast epithelial cell line.

**Methods:**

The in vitro influence of 2-methoxyestradiol-bis-sulphamate was investigated on cell cycle progression, possible induction of apoptosis and autophagy and reactive oxygen species generation. Cell cycle progression was done using flow cytometry in conjunction with ethanol fixation and propidium iodide staining. Displaying effects on the mitochondrial membrane potential was achieved utilizing flow cytometry and the MitoCapture ^TM^ Mitochondrial apoptosis detection kit. Autophagy detection was done by means of flow cytometry and anti-LC3B conjugated to DyLight 488. Reactive oxygen species generation was conducted employing flow cytometry and 2,7-dichlorofluorescein diacetate and hydroethidine.

**Results:**

This study demonstrated that 2-methoxyestradiol-bis-sulphamate did not affect cell cycle progression or reactive oxygen species in a statistically significant manner in the non-tumorigenic MCF-12A cell line. In addition, 2-methoxyestradiol-bis-sulphamate did not statistically significantly induce apoptosis or autophagy.

**Conclusion:**

Reports indicate that 2-methoxyestradiol-bis-sulphamate induces apoptosis and autophagy in several tumorigenic cell lines. The anticancer ability of 2-methoxyestradiol-bis-sulphamate is due to its antimitotic activity. However, this study demonstrates the promising notion that 2-methoxyestradiol-bis-sulphamate does not affect the non-tumorigenic MCF-12A cells. This project contributes to the embedded scientific knowledge regarding the differential death mechanisms used by 2-methoxyestradiol-bis-sulphamate on tumorigenic and non-tumorigenic cell lines.

## Introduction

Researchers have identified a promising anticancer compound, 2-methoxyestradiol (2ME), that exerts antimitotic- and antiproliferative activity in vitro and in vivo [1,2]*.* However, 2ME failed to advance to United States Food and Drug Administration approval due to efficacy concerns [3]. 2ME possesses low bioavailability and rapid metabolic breakdown due to being targeted by the 17β-hydroxysteroid dehydrogenase-mediated metabolism pathway where 2ME is swiftly inactivated by conjugation and oxidation of the hydroxyl groups at the C3/C17 positions [4].

2-Methoxyesradiol-bis-sulphamate is (2MEBM) a bis-sulphamoylated derivative of 2ME that exerts antiproliferative- and anticancer activity with improved bioavailability and a superior pharmacokinetic profile [5-7]. 2MEBM reduced cell growth in a range of cell lines including the estrogen receptor positive breast adenocarcinoma cell line (MCF-7), prostate cancer cell line (LNACaP), estrogen receptor negative breast adenocarcinoma cell line (MDA-MB-231), human cervical adenocarcinoma cell line (HeLa) and human ovarian carcinoma (A2780) [7-12]. In addition, apoptosis was induced by 2MEBM in MCF-7, prostate cancer cells (PC-3), human umbilical vein endothelial cells (HUVEC) and the human breast adenocarcinoma CAL51 cell line [13-15].

Reports have also established that 2MEBM is tenfold more potent than 2ME in its antiproliferative activity [7,16]. The increased potency of sulphamoylated agents are due to their ability to interact with sulphatase, carbonic anhydrase and proteins such as tubulin [17]. In addition, the estrogen 3-*O*-sulphamates strongly inhibit carbonic anhydrase II which is responsible for the conversion of carbon dioxide and water to carbonic acid. This interaction is most likely responsible for the high bioavailability of the sulphamoylated analogues as reversible uptake by red blood cells and interaction with carbonic anhydrase II ensures transiting the liver without undergoing first pass metabolism [1,17,18]. The superior bioavailability of 2MEBM is demonstrated by a previous study where 2MEBM was still detectable in the plasma after 24 h of exposure [7]. Newman, et al. (2006) reported the increased bioavailability of 2MEBM is due to its resistance to metabolism by 17β-hydroxysteriod dehydrogenase type 1 and type 2 enzymes [19].

Anticancer agents ideally induce cell death in tumorigenic cells leaving non-tumorigenic cells unaffected. Thus, determining the influence of 2MEBM on non-tumorigenic cell lines is essential. During this study the in vitro effects of 2MEBM on reactive oxygen species generation and the induction of apoptosis and autophagy in a non-tumorigenic breast epithelial cell line was determined. This study contributes toward the unravelling of differential action mechanisms on tumorigenic and non-tumorigenic cell lines.

## Methods and materials

### Materials

#### Cell line

The MCF-12A cell line is a non-tumorigenic spontaneously immortalized adherent human breast epithelial cell line and forms domes in confluent cultures. The MCF-12A cells were a gift from Professor Parker (Department of Medical Biochemistry, University of Cape Town, South Africa).

### Reagents

All required reagents of cell culture analytical grade were purchased from Sigma (St. Louis, United States of America) unless otherwise specified. Heat-inactivated fetal calf serum (FCS), sterile cell culture flasks and plates were purchased from Sterilab Services (Kempton Park, Johannesburg, South Africa). Penicillin, streptomycin and fungizone were obtained from Highveld Biological Pty (Ltd) (Sandringham, South Africa). The Annexin V fluorescein isothiocyanate (FITC) kit, MitoCapture ^TM^ Mitochondrial apoptosis detection kit and a rabbit polyclonal anti-LC3B conjugated to DyLight 488 were purchased from BIOCOM biotech (Pty) Ltd. (Clubview, South Africa). 2,7-Dichlorofluorescein diacetate and hydroethidine was acquired from Sigma (St. Louis, United States of America). Since 2MEBM is not commercially available, it was synthesized by Professor Vleggaar from the Department of Chemistry (University of Pretoria, Pretoria, South Africa).

### Cell culture

Cells were grown in sterile 25 cm^2^ tissue culture flasks at a humidified atmosphere at 37°C and 5% CO_2_. MCF-12A cells were cultured in medium consisting of a 1:1 mixture of Dulbecco’s minimum essential medium eagle (DMEM) and Ham’s-F12 medium, 20 ng/ml epidermal growth factor, 100 ng/ml cholera toxin, 10 μg/ml insulin and 500 ng/ml hydrocortisone, supplemented with 10% heat-inactivated fetal calf serum (56°C, 30 min), 100U/ml penicillin G, 100 μg/ml streptomycin and fungizone (250 μg/l).

A stock solution of 2x10^-3^ M 2MEBM dissolved in dimethyl sulphoxide (DMSO) was prepared and diluted with medium to the desired concentrations prior to exposure of the cells. Media of all control experiments were supplemented with an equal volume of DMSO (vehicle) with the DMSO content of the final dilutions never exceeding 0.05% (v/v). Experiments entailed seeding 500 000 exponentially growing MCF-12A cells per 25 cm^2^ cell culture flasks containing a final volume of 5 ml of maintenance medium. A 24 h incubation period at 37°C was allowed for cell adherence, the medium was discarded and cells were exposed to 0.4 μM 2MEBM and incubated for 48 h at 37°C. These conditions were selected since previous studies in our laboratory have demonstrated successful antiproliferative activity in tumorigenic cell lines [5,6].

### Methods

#### Cell cycle progression

Flow cytometry was utilized to investigate the DNA content to determine cell cycle distribution, G_2_/M block and the presence of a sub-G_1_ apoptotic peak. After 48 h of exposure to 0.4 μM 2MEBM, cells were trypsinized and resuspended in 1 ml growth medium. 1x10^6^ cells were centrifuged for 5 min at 300xg. The pellet resuspended twice in ice-cold phosphate buffer solution (PBS). The supernatant was discarded and the cells were resuspended in 200 μl of ice-cold PBS containing 0.1% FCS. Ice-cold 70% ethanol (4 ml) was added in a drop wise manner and cells were stored at 4°C for 24 h. After 24 h, cells were pelleted by centrifugation for 5 min. The supernatant was removed and cells were resuspended in 1 ml of PBS containing propidium iodide (40 μg/ml) and incubated at 37°C, 5% CO_2_ for 45 min. Subsequently, cells were analysed by means of FACS FC500 System flow cytometer (Beckman Coulter South Africa (Pty) Ltd) equipped with an air-cooled argon laser excited at 488 nm. Data from at least 10 000 cells were captured with CXP software [Beckman Coulter South Africa (Pty) Ltd, Johannesburg, Gauteng, South Africa] and analyzed with Cyflogic (CyFlo Ltd., Turku, Finland).

### Mitochondrial membrane potential

Mitochondrial integrity was investigated using the MitoCapture ^TM^ mitochondrial kit. The mitochondrial (intrinsic) apoptosis pathway is activated by stress signals that results in the permeabilization of the mitochondrial outer membrane. This will be demonstrated by the MitoCapture ^TM^ mitochondrial kit that supplies quantitive apoptosis data [20]. After 48 h of exposure to 0.4 μM 2MEBM, cells were trypsinized and centrifuged at 13 000 x g. Cells were resuspended in 1 ml MitoCapture solution (1 μl MitoCapture: 1 ml pre-warmed incubation buffer provided by the MitoCapture^TM^ mitochondrial kit), incubated at a humidified atmosphere (37°C, 5% CO_2_) for 20 min and subsequently centrifuged at 500 x g. After the supernatant was discarded, cells were resuspended in 1 ml prewarmed incubation buffer (37°C). Afterwards, cells were analyzed immediately using fluorescence activated cell sorting (FACS) FC500 System flow cytometer (Beckman Coulter South Africa (Pty) Ltd). Apoptotic cells were detected in the FITC channel (usually FL1) showing diffused green fluorescence. Data from at least 10 000 cells were analysed using CXP software [Beckman Coulter South Africa (Pty) Ltd, Johannesburg, Gauteng, South Africa] and analyzed with Cyflogic (CyFlo Ltd., Turku, Finland).

### Autophagy detection

Flow cytometry utilizing the rabbit polyclonal anti-LC3B conjugated to DyLight 488 demonstrated possible autophagy induction. LC3-I is converted to LC3-II by a series of reactions [21]. Enhancement of LC3-I conversion to LC3-II results in the upregulation of autophagy. Thus, detection of LC3-II is a useful indication for the presence of autophagy [22]. After 48 h of exposure to 0.4 μM 2MEBM cells were trypsinised and centrifuged. Cells were washed with cold PBS, pelleted and fixed with 3 ml 0.01% formaldehyde in PBS for 10 min at 4°C. Cells were centrifuged and resuspended in 200 μl PBS, followed by 1 ml ice-cold methanol (−20°C) for 15 min at 4°C. Afterwards cells were washed twice with cold PBS. Cells were stained with 0.5 ml of the antibody cocktail (0.05% Triton X-100, 1% bovine serum albumin (BSA), 40 μg/ml propidium iodide and 0.5 μg/ml conjugated rabbit polyclonal anti-LC3B antibody) prepared in PBS for 2 h at 4°C. Cells were washed trice with PBS/0.05% Triton/1% BSA and analyzed by means of flow cytometry. Data from at least 10 000 cells were analyzed employing Cyflogic version 1.2.1 software (Pertu Therho, Turko, Finland).

### Reactive oxygen species generation

Hydrogen peroxide (H_2_O_2_) generation was assessed using 2,7-dichlorofluorescein diacetate (DCFDA), a non-fluorescent probe, which, upon oxidation by ROS and peroxides is converted to the highly fluorescent derivative 2,7-dichlorofluorescein (DCF). Superoxide generation was assessed using hydroethidine (HE) that is oxidized by superoxide, to a red fluorescent compound. After 0.4 μM 2MEBM exposure for 48 h exposure, cells were trypsinized and 1x10^6^ cells were resuspended in 1 ml PBS and washed with PBS. Cells were incubated with 20 μM DCFDA for 25 min and 10 μM HE for 15 min at 37°C. DCF (FL1) and HE fluorescent product fluorescence (FL2) were measured with a FACS FC500 System flow cytometer equipped with an air-cooled argon laser excited at 488 nm. The information generated from at least 10 000 cells were analyzed by means of Cyflogic version 1.2.1 software (Pertu Therho, Turko, Finland).

### Statistics

Measurement of FITC-, HE- and DCF-derived fluorescence was expressed as a ratio of the value measured for the 2MEBM-treated cells compared to vehicle-treated exposed cells (mean relative fluorescence). Flow cytometry analysis involved data from at least 10 000 events that was repeated thrice where after a representative figure was chosen for each experiment. Flow cytometry data were analyzed by means of Cyflogic version 1.2.1 software (Pertu Therho, Turko, Finland).

## Results

### Cell cycle progression

**Figure 1:**
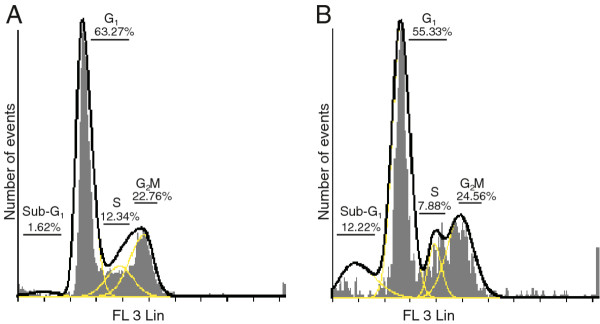
** Flow cytometry demonstrated cell cycle distribution of vehicle-treated cells (A) and 2MEBM-treated (B) MCF-12A cells.** The sub-G_1_ fraction did increase non-statistically significantly 10% accompanied with a decrease in G_1_-phase when compared to vehicle-treated cells.

Flow cytometry and propidium iodide was employed to demonstrate the effects of 2MEBM on cell cycle progression and possible apoptosis induction (Figure 1). A statistically insignificant 10% increase was demonstrated in the sub-G_1_ fraction after 48 h exposure to 0.4 μM 2MEBM accompanied with a decrease in the G_1_ phase. No statistically significant changes were indicated in any cell cycle phase when compared to the vehicle-treated cells.

### Mitochondrial membrane potential

**Figure 2:**
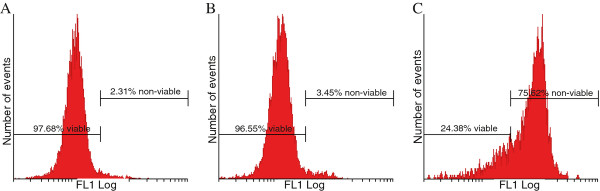
** Permeabilization of the outer mitochondrial membrane of vehicle-treated (A), 2MEBM-treated MCF-12A cells (B) and MCF-12A cells treated with Actinomycin D (0.1 μg/ml) (positive control for apoptosis induction) (C) were displayed using flow cytometry and the MitoCapture**^**TM**^**Mitochondrial apoptosis detection kit.** Mitochondrial membrane potential was statistically insignificantly affected in 3.45% when compared to 2.31% vehicle-treated cells.

Data was obtained regarding the mitochondrial membrane permeabilization using the MitoCapture ^TM^ mitochondrial kit and flow cytometry (Figure 2). A statistically insignificant 1% increase was observed in the amount of 2MEBM- treated cells with a reduced mitochondrial membrane potential when compared to vehicle-treated cells.

### Autophagy detection

**Figure 3:**
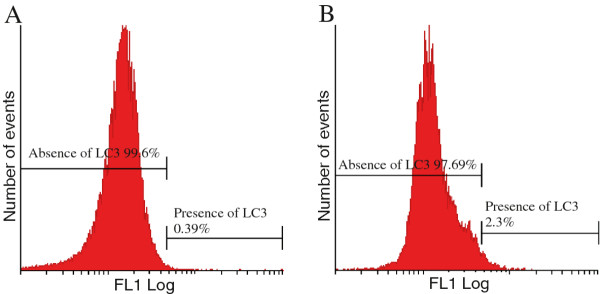
Flow cytometrical investigation employing the conjugated anti-LC3 antibody to investigate autophagy induction in vehicle-treated cells (A) and 2MEBM-treated cells (B) revealed that 2.3% of 2MEBM treated MCF-12A cells were present in autophagy when compared to 0.39% of vehicle-treated cells.

A conjugated rabbit polyclonal anti-LC3B antibody employing flow cytometry was utilized for the detection of autophagy (Figure 3). The latter demonstrated the accumulation of LC3 in 2.3% of the 2MEBM -treated cells when compared to 0.4% of the vehicle-treated cells.

### Reactive oxygen species generation

**Figure 4:**
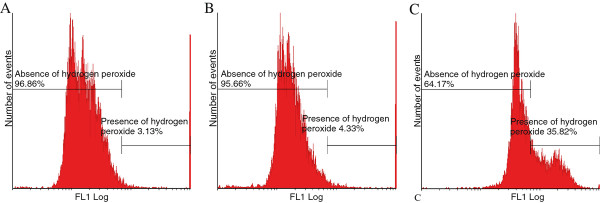
** Flow cytometry in conjunction with demonstrating hydrogen peroxide and 2,7-dichlorofluorescein diacetate of vehicle-treated (A), 2MEBM-treated MCF-12A cells (B) and cells treated with 20 μM hydrogen peroxide.** The latter served as a positive control for hydrogen peroxide generation (**C**). A statistically insignificant 1% of cells had increased hydrogen peroxide in 2MEBM cells when compared to vehicle-treated cells.

**Figure 5:**
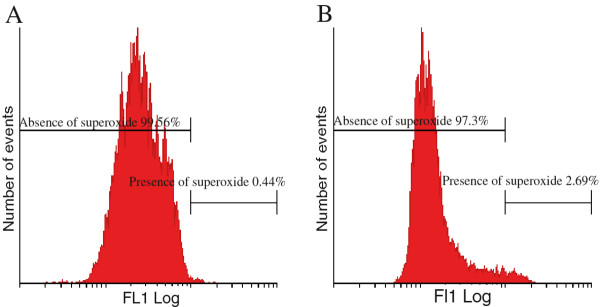
** Superoxide generation investigation using flow cytometry and hydroethidine of vehicle-treated (A) and 2MEBM-treated MCF-12A cells (B).** An increase of 2% was found in the 2MEBM-treated cells when compared to the vehicle-treated cells.

Flow cytometry employing DCFDA and HE demonstrated the in vitro effect of 2MEBM on hydrogen peroxide and superoxide production, respectively (Figure 4 and Figure 5). Statistically insignificant increases were found in hydrogen peroxide and superoxide in 2MEBM-treated cells when compared to vehicle-treated cells.

## Discussion

During the investigation of a compound’s potential anticancer activity it is crucial to scrutinize the effects on non-tumorigenic cell lines as it is essential that the compound leaves non-tumorigenic cells less-or unaffected. As previously mentioned 2MEBM exerts antiproliferative activity and demonstrates potential as an anticancer agent [5,6]. This in vitro study explored the effects of 2MEBM on cell cycle progression and cell death induction in a non-tumorigenic breast epithelial cell line.

Various reports have indicated that 2MEBM exerts an antimitotic effects and induced a G_2_/M block in several cell lines including drug resistant human breast adenocarcinoma cell line (MCF-7 _DOX_40), mitoxantrone resistant breast adenocarcinoma cell line (MCF-7 MR) and MDA-MB-231 [9,12]. Cell cycle progression analysis from the current study revealed that 2MEBM did not affect the G_2_/M fraction. In addition, the statistically insignificant sub-G_1_ fraction in the current study demonstrates that 2MEBM does affect non-tumorigenic cell lines less as 0.4 μM 2MEBM exposure for 48 hours resulted in apoptosis induction in 50% of MCF-7 cells [5,6]. The notion that 2MEBM affects non-tumorigenic cell lines less with regard to apoptosis induction is verified by the MitoCapture ^TM^ mitochondrial results that indicate 2MEBM has no significant effects the mitochondrial membrane potential.

Previously published novel findings indicate that 2MEBM induces autophagy in MCF-7 cells and the oesophageal tumorigenic cell line (SNO) [6,23]. However, Vorster, et al. (2010) reported that 2MEBM did not induce autophagy in the non-tumorigenic cell line [24]. The latter is supported in this study where 2MEBM failed to induce autophagy in the MCF-12A cells.

The increased reactive oxygen species production after exposure to 2MEBM in MCF-7 cells was recently reported [25]. The current study revealed the novel finding that 2MEBM exposure did not result in a significantly significant increase in hydrogen peroxide or hydrogen peroxide production. Studies indicate that reactive oxygen species is associated with apoptosis and autophagy [26]. Reactive oxygen species may be involved in the direct activation of death receptors and reactive oxygen species-induced receptor clustering and signaling by means of the lipid raft platforms; however, this suggestion is novel and requires research [27]. In addition, increased reactive oxygen species production results in permeabilization of the mitochondrial outer membrane and subsequent cytochrome *c* release leading to apoptosis [27]. In addition, Shrotriya*,* et al. (2012) reposted that reactive oxygen species generation resulted in apoptosis induction in neck sqamous carcinoma cells [28]. Recent reports indicate that reactive oxygen species involved with autophagy induction using several mechanisms including Atg4, catalase and the mitochondrial electron transport chain resulting in both cell survival and cell death [29]. Reactive oxygen species production results in Beclin-1 overexpression resulting in autophagy induction. Hydrogen peroxide is necessary for starvation-induced autophagy and directly targets Atg4 for oxidation and inactivates Atg4 inducing autophagy [25].

In conclusion, this promising study revealed evidence that 2MEBM does not affect the non-tumorigenic MCF-12A cells in a statistically significant manner. Thus, the latter indicates that 2MEBM influences tumorigenic and non-tumorigenic cell lines differentially as 2MEBM is a well-reported compound with antiproliferative activity that induces cell death [5,6]. This study contributes to elucidating the differential mechanism of action used by 2MEBM in tumorigenic and non-tumorigenic cell lines.

## Competing interests

The authors declare there are not any competing interests.

## Authors’ contribution

MH Visagie and AM Joubert conducted the project design. MH Visagie conducted the experiments and data analysis and compiled the manuscript. AM Joubert managed the funding acquisition from various grants, supervised the project and was involved in interpretation of data and revision of the manuscript. All authors have contributed and approved the final manuscript.
